# Chiral Peptide Nucleic Acids with a Substituent in the *N*-(2-Aminoethy)glycine Backbone

**DOI:** 10.3390/molecules18010287

**Published:** 2012-12-27

**Authors:** Toru Sugiyama, Atsushi Kittaka

**Affiliations:** 1 Department of Life Sciences, Graduate School of Arts and Sciences, The University of Tokyo, Komaba, Meguro-ku, Tokyo 153-8902, Japan; 2 Faculty of Pharmaceutical Sciences, Teikyo University, Kaga, Itabashi-ku, Tokyo 173-8605, Japan; E-Mail: akittaka@pharm.teikyo-u.ac.jp

**Keywords:** peptide nucleic acid, chiral, preorganization, antigene

## Abstract

A peptide nucleic acid (PNA) is a synthetic nucleic acid mimic in which the sugar-phosphate backbone is replaced by a peptide backbone. PNAs hybridize to complementary DNA and RNA with higher affinity and superior sequence selectivity compared to DNA. PNAs are resistant to nucleases and proteases and have a low affinity for proteins. These properties make PNAs an attractive agent for biological and medical applications. To improve the antisense and antigene properties of PNAs, many backbone modifications of PNAs have been explored under the concept of preorganization. This review focuses on chiral PNAs bearing a substituent in the *N*-(2-aminoethyl)glycine backbone. Syntheses, properties, and applications of chiral PNAs are described.

## 1. Introduction

**Figure 1:**
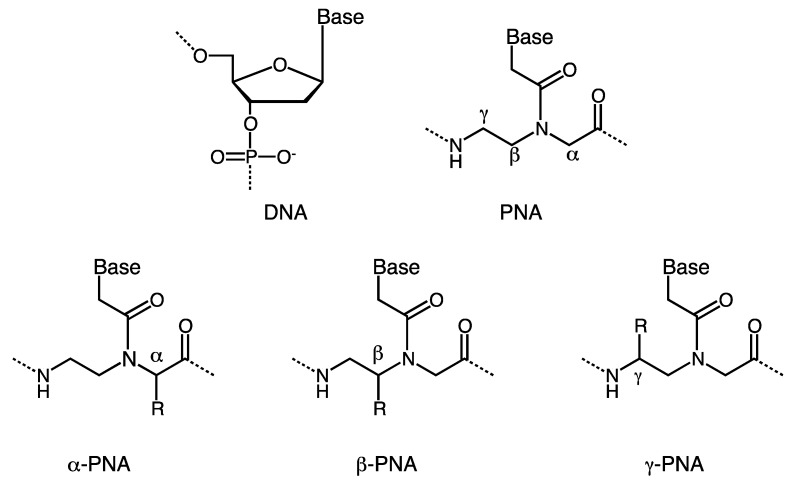
Chemical structures of DNA, PNA, and chiral (α-,β-,γ-)PNAs.

A peptide nucleic acid (PNA) is a synthetic analogue of DNA first reported by Nielsen *et al*. in 1991 [1]. In PNA, the sugar-phosphate backbone of DNA is replaced by a peptide backbone consisting of *N*-(2-aminoethyl)glycine units. PNA oligomers hybridize to complementary sequences by Watson-Crick base pairing in spite of its structural difference from DNA [2,3,4]. Since the neutral peptide backbone of PNA does not have electrostatic repulsion that generally destabilizes DNA-DNA duplexes, thermal stability of PNA-DNA duplexes is higher than that of DNA-DNA duplexes. The relative rigidity of the PNA backbone improves sequence selectivity on hybridization. PNAs are resistant to nucleases and proteases [5] and have a low affinity for proteins [6]. A remarkable feature of PNAs is their ability to invade double-stranded DNA. Some reports suggest that PNAs can target duplex DNA even in living cells by strand invasion [7,8,9,10,11]. Strand invasion requires high stability of PNA-DNA duplexes to compete with the displaced DNA strand. To improve the antisense and antigene potency of PNAs, it is reasonable to increase binding affinity for DNA and RNA by suitable preorganization. An approach for improving DNA binding affinity is the design and synthesis of preorganized PNAs preferring a right-handed helical conformation. Reports have appeared demonstrating that preorganization can be achieved by cyclization of the PNA backbone or by adding substituents to the backbone [12,13]. In addition, original PNA has some drawbacks such as poor cellular uptake and relatively low solubility in aqueous media. The development of modified PNAs also aims to overcome these problems. In the present review, we intend to focus on chiral PNAs bearing substituents on the original *N*-(2-aminoethyl)glycine backbone and present them divided into three groups (α-PNA, β-PNA, and γ-PNA) based on the position of a substituent in the PNA backbone (Figure 1).

## 2. α-PNA

### 2.1. Synthesis and Properties of α-PNAs

Nielsen *et al*. reported the first α-chiral PNA in 1994, in which glycine moiety of the PNA backbone was substituted by alanine [14]. Both the L- and D-forms of the chiral monomers were synthesized from L- or D-alanine and incorporated into oligomers. Thermal stability of PNA-DNA duplex containing D-form monomers was similar to that of the original PNA with a glycine backbone, whereas a reduction was observed when L-form monomers were incorporated. Reductive amination is the most used procedure to obtain an α-chiral aminoethyglycine backbone and a variety of α-chiral PNAs bearing side chains from amino acids have been prepared by this method [15,16,17]. Alternatively, solid-phase synthesis of chiral monomers has been reported [18].

A critical problem in the synthesis of chiral α-PNAs is racemization of α-carbon during oligomer synthesis [19]. A systematic study of the optical purity of PNA oligomers as a function of the coupling conditions used in solid-phase synthesis revealed that DIC/HOBt gives the best result. Coupling with HATU or HBTU as a coupling agent resulted in relatively high levels of racemization. In particular, HATU gave rise to more rapid racemization [20]. Since HATU and HBTU are widely used in PNA synthesis, these results have to be taken into account. The optical purity of α-PNA oligomers can be improved by omitting the preactivation step when using HATU.

**Scheme 1:**
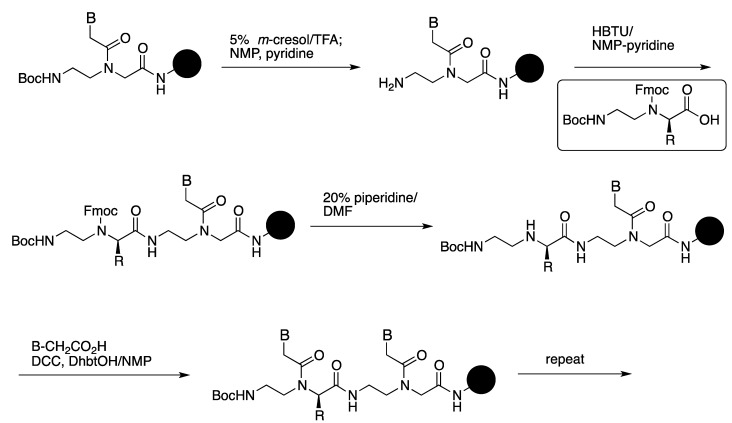
Submonomeric cycle for the insertion of a chiral monomer into a PNA chain on resin [23].

Submonomer solid-phase synthesis has been reported as an alternative method for the synthesis of PNAs [21,22]. Sforza *et al*. developed a submonomeric approach to minimize the racemization of PNA during the solid-phase synthesis [23]. An orthogonally protected chiral submonomer (boxed in Scheme 1) was used for Boc-based solid-phase synthesis. A chiral PNA monomer unit was constructed by Fmoc deprotection of the submonomer and coupling with the carboxymethylnucleobase on resin (Scheme 1). This approach gives α-PNA oligomers with the highest optical purity among reported methods.

Introduction of a side chain at the α-position caused moderate destabilization of PNA-DNA duplexes relative to the unmodified PNA [14,15,16,24]. The extent was larger for PNA oligomers carrying L-form monomers than for those carrying D-form monomers. In contrast, D-Lys-based PNA exhibited higher *T*_m_ than that of unmodified PNA. This was due to electrostatic attraction between the ammonium group of lysine side chain and the phosphate groups of DNA. 

Incorporation of three consecutive D-Lys-based α-PNA monomers greatly improved the sequence selectivity of PNA oligomer with moderate expense to DNA binding affinity (Table 1) [25] and the crystal structure of a PNA decamer containing three D-Lys-based monomers hybridized with its complementary DNA provided valuable insights into the PNA structure. Crystallographic data indicate that α-PNAs prefer the P-helix conformation. The introduction of a chiral center at the α-position of the PNA backbone limits the ability of the PNA strands to adopt other conformations, thus improving the mismatch discrimination ability [26].

**Table 1:** Melting temperatures (°C) of D-lysine-based α-PNA-DNA duplexes [25].

PNA ^a^	*T*_m_ (°C)	*T*_m _(°C)
	perfect match	single mismatch
	5'-AGTGATCTAC-3'	5'-AGTG*G*TCTAC-3'
H-GTAGATCACT-NH_2_	50	40
H-GTAG**ATC**ACT-NH_2_	43	-
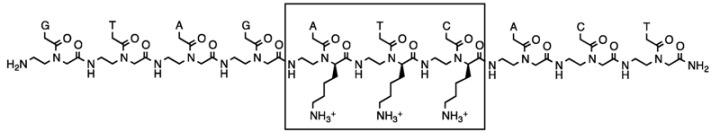		

^a^ Bold letters indicate modified backbone units.

**Figure 2:**
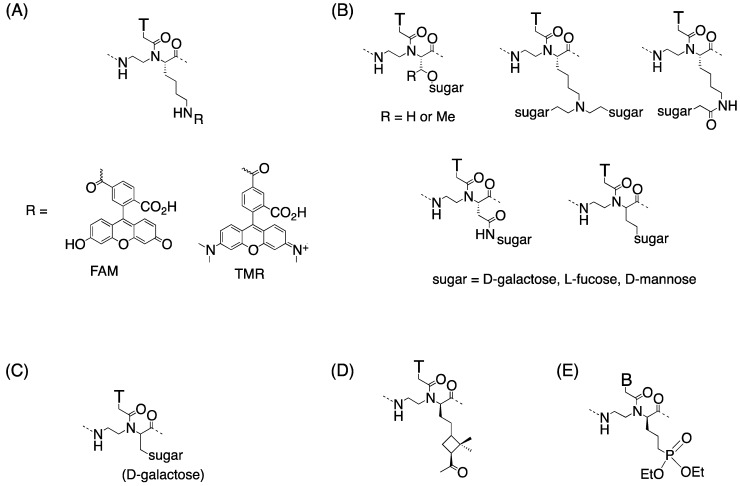
Structures of α-PNAs bearing functional molecules [27,30,31,32,33].

The lysine side chain of α-Lys monomer was also used as a handle for site-specific incorporation of functional molecules into PNA oligomers. For example, Seitz *et al*. synthesized PNAs carrying FAM and TMR fluorophores in the middle of the PNA sequences (Figure 2A) [27].

D-arginine-based PNA is a special case (Table 2). Although the guanidinium group of α-Arg PNA (α-GPNA) is positively charged under physiological pH, incorporation of one unit of α-GPNA into PNA decamer destabilized the PNA-DNA duplex; however, incorporation of multiple units of D-form α-GPNAs at every other position improved DNA binding affinity. By spacing the arginine units, electrostatic attraction between the guanidinium groups and the phosphates seems to overcome the adverse effect of steric repulsion arising from arginine side chains themselves [28]. Recently, α-GPNA was used for PNA microarray and its higher sequence selectivity has been demonstrated [29].

**Table 2:** Melting temperatures (°C) of α-GPNA-DNA duplexes [28]^a^.

	Sequence	*T_m_* (°C)	*T_m_*
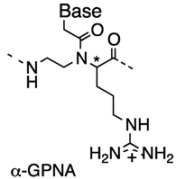	H-GCATGTTTGA-^L^Lys-NH_2_	43	
	H-GCATG^L^**T**TTGA-^L^Lys-NH_2_	39	−4
	H-GCATG^D^**T**TTGA-^L^Lys-NH_2_	41	−2
	H-GCA^D^**T**G^D^**T**T^D^**T**GA-^L^Lys-NH_2_	47	+4
	H-G^D^CA^D^**T**G^D^**T**T^D^**T**G^D^A-^L^Lys-NH_2_	50	+7

^a^ Bold letters indicate modified backbone units and superscripts (L or D) indicate the configuration of the amino acids from which the backbone units were derived.

As expected, the introduction of negatively charged side chains into the backbone destabilized PNA-DNA duplexes [15]. Moreover, PNAs derived from bulky amino acids, such as Tyr, His, Trp, Phe and Val, showed lower *T*_m_s due to steric hindrance [16].

Nielsen *et al.* reported the synthesis of a series of α-PNAs bearing glycosylated side chains at the α-position and demonstrated their selective biodistribution (Figure 2B) [30]. Metzler-Nolte *et al*. reported the synthesis of *C*-linked glycosylated α-PNA and its successful incorporation into a PNA oligomer using Fmoc chemistry (Figure 2C) [31]. In addition to natural amino acids and carbohydrates, cyclobutyl-carbonyl-containing α-PNA monomer has been stereoselectively synthesized from (+)-α-pinene and its incorporation did not change the binding affinity of the PNA oligomer towards RNA, DNA or PNA with the complementary sequence (Figure 2D) [32]. Czerny *et al*. recently introduced phosphonic ester into the α-side chain (pePNA) (Figure 2E). Its incorporation into standard PNA oligomers improved antisense potency compared to unmodified PNAs [33].

**Figure 3:**
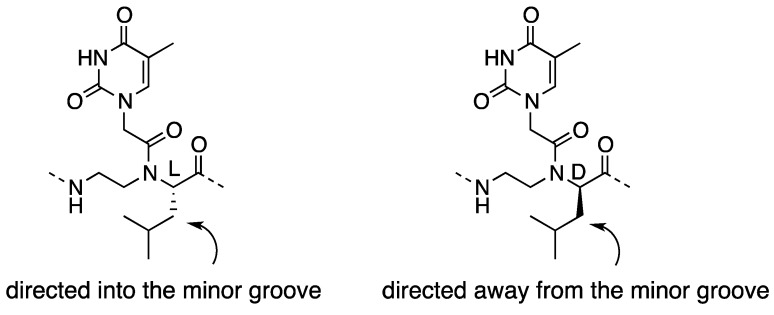
Structures of L- or D-leucine-based α-PNAs [34].

Armitage *et al*. used α-PNAs as a tool for studying interactions between small molecules and the PNA-DNA duplex [34]. They synthesized PNA oligomers containing L- or D-leucine-based α-PNA monomers (Figure 3) and compared the effect of α-side chains on cyanine dye aggregation. Incorporation of L-luecine-based units significantly hindered cyanine dye aggregation, whereas the D-leucine analogue was less inhibitory. Since isobutyl groups project into the minor groove in the L-leucine-based duplex, effective blockage of the dye access suggests minor groove binding of the dye. The authors proposed a molecular recognition mechanism in which cyanine dyes assemble into helical aggregates by using the minor groove of a PNA-DNA duplex as a template. 

**Figure 4:**
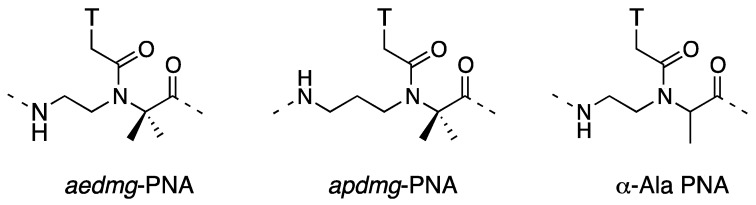
Structures of α,α-dimethyl PNAs [35] and alanine-based α-PNA.

Although achiral, we would also like to mention α,α-disubstituted PNA (Figure 4). Recently, Ganesh *et al*. reported that the incorporation of *gem*-dimethyl substituted PNA monomers into PNA oligomers increases the *T*_m_ of PNA-DNA duplexes [35]. Interestingly, incorporation of the homologous aminopropyl-(α,α-dimethyl)glycyl (*apdmg*)-PNA monomers also improved DNA binding. It would be intriguing to compare this finding with alanine-based α-PNA whose incorporation into the oligomer does not significantly change the thermal stability of PNA-DNA duplexes [14]. Introduction of appropriate rigidity without any chirality improved DNA binding properties of PNA. Another interesting feature of dimethyl PNAs is their preferential binding to DNA than to RNA.

### 2.2. Double-Duplex Invasion

**Figure 5:**
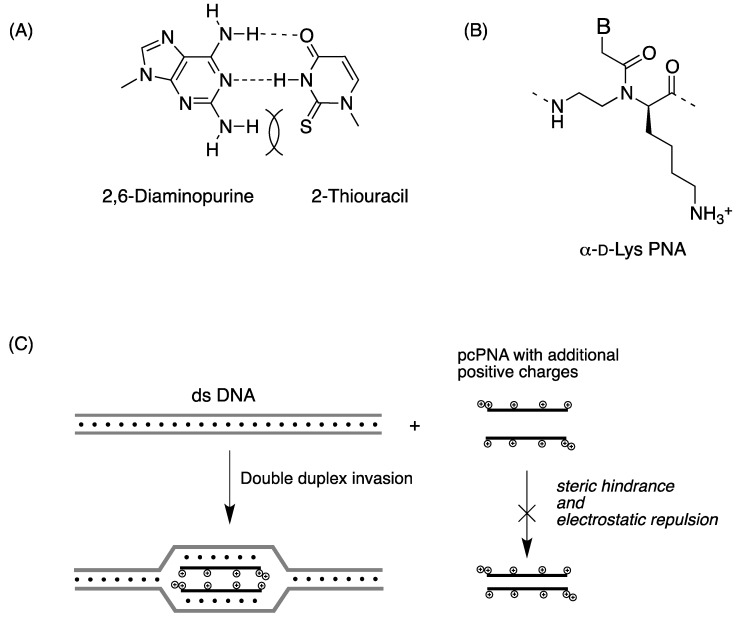
(**A**) Pseudo-complementary bases 2,6-diaminopurine and 2-thiouracil used in pcPNA; (**B**) D-lysine-base α-PNA; (**C**) Illustration of double-duplex invasion process of pcPNAs with additional positive charges. Electrostatic repulsion further destabilized the PNA-PNA duplex [36,37].

Double-duplex invasion is a binding mode of PNA that targets both DNA strands simultaneously using pseudocomplementary PNA (pcPNA) pairs [36]. In pcPNA, adenine and thymine were replaced with 2,6-diaminopurine and 2-thiouracil, respectively (Figure 5A). The diaminopurine-thiouracil base pair is significantly destabilized because of steric hindrance, and thus, PNA-PNA duplex formed by pcPNA strands became unstable, while PNA-DNA duplexes retained high stability. The efficiency of double-duplex invasion was dependent on A/T contents of target DNA sequences and the invasion did not proceed efficiently when G/C rich sequences were targeted. In 2008, Komiyama *et al*. incorporated positive charges into pcPNA pairs by using α-D-Lys PNA monomers (Figure 5B) [37]. In this system PNA-PNA duplex is further destabilized by additional electrostatic repulsion and, as a result, even G/C rich sequences could be targeted. Finally, this approach reduced the sequence requirement for efficient double-duplex invasion from 40% A/T content to 20%.

### 2.3. Cellular Uptake of α-PNAs

Considered as an antisense or antigene agent, poor cellular uptake of PNA is a significant drawback and several methods have been developed to overcome this problem [38]. These include conjugation of PNAs to cell-penetrating peptides (CPPs) and chemical modification of the PNA backbone. PNA-peptide conjugates can be internalized into cells through endocytosis [39]. The major fraction of PNA localizes to endosomal compartments and is not available for DNA/RNA targeting. Thus, only a small fraction of PNA that escapes the endosome or is taken up by alternative pathways can exhibit biological activity. To promote the release of PNA-peptide conjugates from endosomes, additives such as calcium ions and chloroquin can be used; however, the potency of PNA-peptide conjugates was improved to only a small extent and these agents exhibited pronounced toxicity [40,41]. Moreover, a 14-mer PNA oligomer containing three units of D-Lys-based α-PNA monomers and its peptide conjugates have been tested for cellular uptake and found to induce endocytotic uptake in most of the tested cell lines [42].

**Figure 6:**
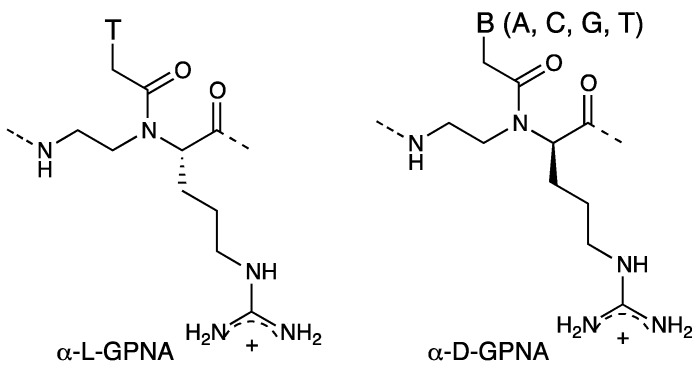
Structures of α-L-GPNA [43] and α-D-GPNA [47].

Ly *et al*. introduced a guanidinium group into the PNA backbone [43] based on the finding that the HIV-1 Tat transcription domain (GRKKRRQRRR) [44], arginine-rich peptides [45], and a homoarginine peptoid exhibited marked cellular uptake properties [46]. Cellular uptake of fully modified α-GPNA decamer derived from L-arginine (α-L-GPNA in Figure 6) was evaluated with human HCT116 (colon) and Sao-2 (osteosarcoma) cell lines. α-GPNA permeated the cell membrane and appeared to localize specifically in the nucleus. Since there was no difference in the uptake properties of α-GPNA at 37 °C and 4 °C, the uptake mechanism of α-GPNA was neither endocytosis-driven nor receptor mediated. Membrane flipping has been proposed as a mechanism [43].

α-GPNA oligomers in which unmodified PNA units and α-GPNA units were alternated at every other position possessed optimal hybridization and cellular uptake properties. Cellular uptake of α-GPNA octamer and dodecamer containing alternate backbone derived from D-arginine (α-D-GPNA in Figure 6) was also addressed and found to be efficient for HeLa and ES cells [47]. In contrast, neither the peptide containing the corresponding number of arginine residues (Fl-abu-RRRR-NH_2_) nor the standard achiral PNA octamer with an *N*-terminal arginine tetramer (Fl-abu-RRRR-CGATCTGA-NH_2_) was taken up by either cell type. Successful cellular uptake of α-GPNA by ES cells is a particularly important finding because ES cells are extremely difficult to transduce, even when the best transfecting agents are used. The authors noticed that their results were consistent with a voltage-driven mechanism of arginine-rich peptides proposed by Wender *et al.* [48]; that is, positively charged guanidinium groups of arginine form bidentate hydrogen bonds with acceptor functionalities on the cell surface such as phospholipids. The resultant ion pair complexes migrate across the cell membrane driven by the difference in the membrane potential and then the peptide enters the cytosol. The same uptake mechanism has been supposed for α-GPNA.

To further gain insight into the uptake properties, the intracellular distribution of a 18-mer, alternating α-D-GPNA oligomer was examined [28]. The α-GPNA was taken up by Hela cells and localized specifically to the endoplamic reticulum (ER) where mRNAs are translated into proteins. This ER-specific localization was distinct from shorter α-GPNAs [47]. Since mRNAs are translated into proteins in ER, specific localization of α-GPNA assured its promising potential as an antisense agent. 

18-mer, α-GPNAs with the same modification were applied to antisense inhibition of human E-cadherin gene expression [49]. Of the six α-GPNA oligomers designed to target the E-cadherin gene transcript, the oligomer designed to bind to the transcription start-site was the most potent. This is consistent with the finding obtained through antisense experiments with achiral PNA oligomers [50]. It is noteworthy that α-GPNAs were less toxic to the cells than the corresponding PNA-polyarginine conjugates. Even at concentrations (10–15 μM) where more than 90% of the cells treated with the PNA-polyarginine conjugate underwent apoptosis, the α-GPNA showed no noticeable sign of toxicity. It has been suggested that the cytotoxic effects of the PNA-peptide conjugates might be due to their amphipathic nature. The hydrophilic polyarginine domain of the conjugate interacts with the phospholipids on the cell surface, while the relatively hydrophobic PNA domain inserts into the lipid bilayers. As a result, the cell membrane may be perturbed, leading to cell death. Meanwhile, α-GPNAs are less amphipathic and therefore less toxic to the cells.

## 3. γ-PNA

### 3.1. Synthesis and Properties of γ-PNAs

**Scheme 2:**
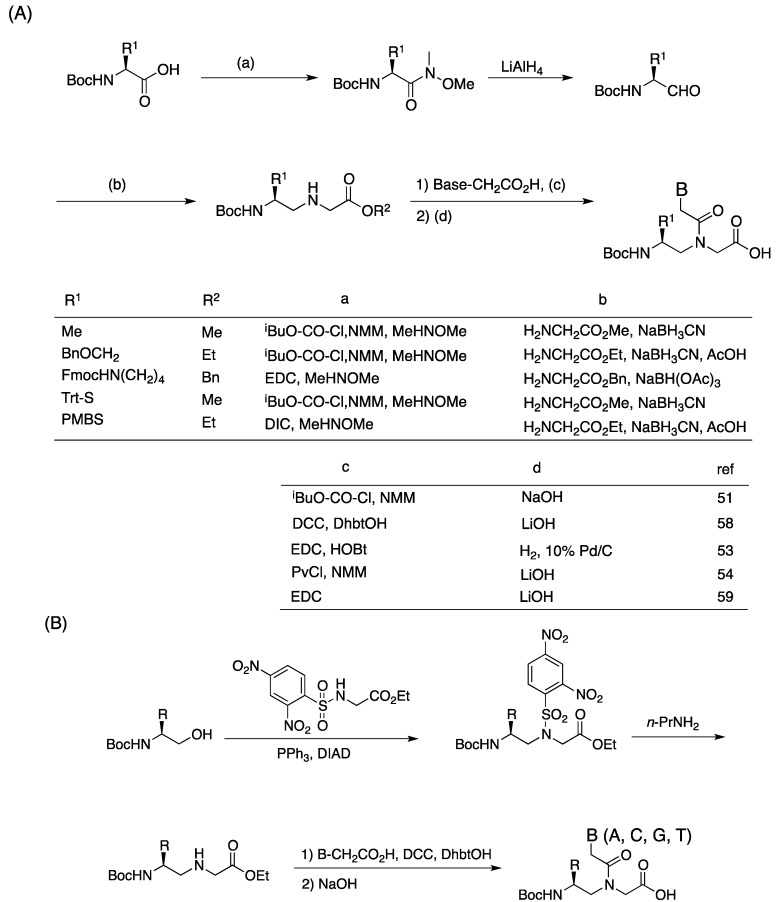
Synthesis of γ-PNA monomer by (**A**) reductive amination [51,53,54,58,59] or by (**B**) Mitsunobu-Fukuyama reaction as a key step [60,61].

Although the first γ-chiral PNA monomer was reported in 1994 [51], oligomers carrying γ-chiral units did not appear until 2005 [52,53,54,55]. As in the case of α-PNAs, most γ-chiral aminoethyglycine backbones were constructed by using reductive amination as a key step (Scheme 2A). Optically active aminoaldehydes are prepared from *N,O*-dimethylhydroxyl amide derivatives of the corresponding amino acids by reduction with LiAlH_4_. Reductive amination of the aldehyde with a glycine ester gave the γ-modified PNA backbone; however, these aldehydes required cautious treatment because they were prone to racemization [56,57]. Using this procedure, various γ-PNA monomers were synthesized, including Ala, Ser, Lys and Cys-based PNAs. In addition, the submonomeric approach that was originally developed for the synthesis of α-PNA was also adopted for γ-PNAs [52].

Some γ-PNA monomers were prepared by the Mitsunobu-Fukuyama reaction (Scheme 2B) [60,61,62]. This procedure used an aminoalcohol as the key intermediate instead of the aminoaldehyde described above. Since the α-proton of aminoalcohol was inert to deprotonation by the base, enantiomerically pure monomers could be obtained. Mitsunobu-Fukuyama coupling of protected aminoalcohol with 2-(2,4-dinitrophenylsulfonamido)acetate proceeded without the risk of racemization. Removal of the dinitrosulfonate protecting group with a mild base yielded a chiral γ-PNA backbone. DCC-mediated coupling with the carboxymethylnucleobases and hydrolysis of the esters gave the γ-PNA monomers.

**Scheme 3:**
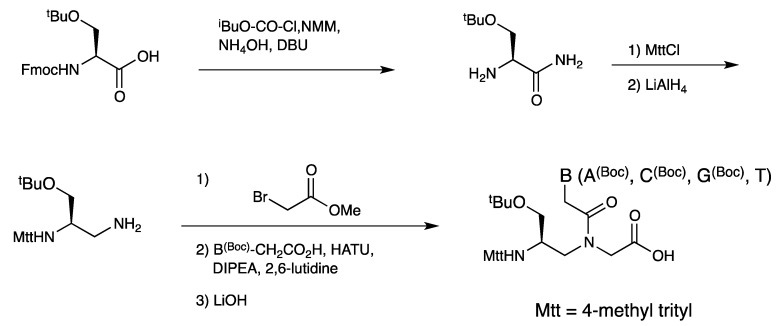
Synthesis of γ-PNA monomers with Mtt/Boc protecting group combination [63].

Winssinger *et al*. reported a protecting group combination (Mtt/Boc) that was orthogonal to Fmoc-based solid-phase PNA synthesis (Scheme 3) [63]. This protocol was developed for PNA-encoded diversity-oriented synthesis, and they synthesized serine-based γ-PNA monomers with these protecting groups. Amine protection of serine amide derived from Fmoc-Ser(*t*Bu)-OH and subsequent reduction with LiAlH_4_ afforded Mtt-protected diamine. Alkylation, acylation and hydrolysis gave γ-PNA monomers protected with Mtt/Boc combination.

**Figure 7:**
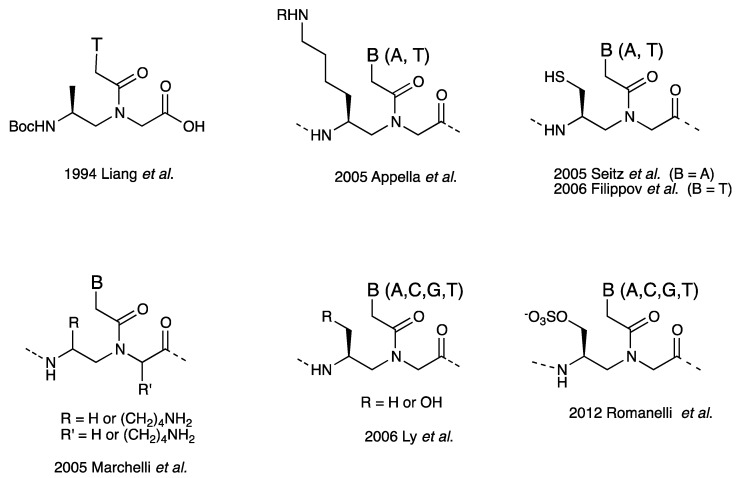
Structures of γ-PNAs [51,52,53,54,64,67,68].

Figure 7 shows the structures of some representative γ-PNAs. In 2005, Appella *et al*. reported PNA oligomers carrying γ-Lys PNA as a handle for fluorene incorporation aiming at developing quencher-free molecular PNA beacons [53]. Incorporation of γ-modified PNA slightly stabilized PNA-DNA duplexes relative to the unmodified duplex. Moreover, the ability of γ-PNA to discriminate single-base mismatches was superior to that of the corresponding unmodified PNA. In the subsequent paper [64], the authors demonstrated that γ-Lys PNA was a versatile scaffold to attach various functional molecules including peptides and fluorophores. UV melting experiments revealed that γ-L-Lys PNA monomer stabilized PNA-DNA duplex, whereas γ-D-Lys PNA monomer markedly destabilized the duplex (Table 3). Recently the authors have applied γ-L-Lys PNAs to multivalent display of integrin ligands in a controlled manner [65]. Cysteine-base γ-PNAs heve been also reported [54,59]. PNA oligomers carrying the Cys-based monomer at the *N*-terminus could be used for native chemical PNA ligation with thioesters of PNAs to yield long chain PNAs. Previous to the report of γ-Lys PNA, γ-Cys PNA was used for the conjugation of functional molecules and such conjugates were used for multivalent scaffold assembly [66]. Marchelli *et al*. compared the thermal stability of α-Lys PNAs with γ-Lys PNAs and revealed that γ-modification was more effective to improve the DNA binding ability. Moreover, the effect of stereochemistry on DNA binding was more evident in γ-PNA than α-PNA [52,67]. Romanelli *et al*. reported γ-PNA bearing a sulfate group aimed at making PNAs more DNA-like in terms of polarity and charge. Three sulfate monomers were incorporated into homopyrimidine PNA nonamer (CTCCTCCTC, in the sequence all Ts were replaced with sulphate T). PNA_2_•DNA triplex formed by sulfate-modified PNA was less stable (Δ*T*_m_ −5.6 °C) than the standard PNA hybrid. This destabilization was explained by electrostatic repulsion between the negatively charged phosphate of DNA and sulphate of the modified PNA. The modified PNA could be lipofected into human breast cancer (SKB3) cells due to its negative charge and exhibited antigene activity against *ErbB2* gene [68].

Detailed spectroscopic studies of serine- or alanine-based γ-PNAs carried out by Ly *et al*. revealed that a simple γ-backbone modification preorganized single-stranded PNA oligomers into a right-handed helical structure that was very similar to that of PNA-DNA duplex. The γ-PNAs bound to DNA with very high affinity and high sequence selectivity. Helical induction was sterically driven and stabilized by base stacking. The fully γ-modified decameric PNA formed exceptionally stable PNA-DNA duplex with an increase of 19 °C of the *T*_m_ compared to the unmodified PNA [58]. The author’s group also reported the crystal structure of a PNA-DNA duplex with complete γ-backbone modification. The structure illustrates that γ-PNA possesses conformational flexibility while maintaining sufficient structural integrity to adopt the P-helical conformation on hybridization with DNA [69]. It is noteworthy that γ-PNAs in the single-strand state (determined by NMR) and in the hybrid duplex state (determined by X-ray crystallography) adopt a very similar conformation.

Ly *et al*. have reported a variety of γ-PNAs bearing side chains derived from amino acids [70]. It was demonstrated that the γ-position could accommodate various hindered side chains without inducing adverse effects on the hybridization properties of PNAs (Table 3). This is in contrast to α-PNA, which is sensitive to steric hindrance arising from side chains at the α-position. Introduction of each chiral unit into the oligomer resulted in an increase in the *T*_m_ of the PNA-DNA duplex by ~4 °C [52,62,68,70].

**Table 3:** Melting temperatures (°C) of γ-PNA-DNA duplexes.

Backbone Modification	*T_m_* (°C)	PNA sequence	Reference
			
Gly	47	a	[70]
L-Ala	51	a	
L-Val	51	a	
L-Ile	51	a	
L-Phe	51	a	
Gly	44	a	[68]
L-Ser	48	a	
Gly	49.7	b	[62]
L-Lys	51.4	b	
D-Lys	36.4	b	
Gly	50	c	[52]
L-Lys	56	c	
D-Lys	32	C	

The PNA sequence is (a) H-GCATG**T**TTGA-^L^Lys-NH_2_, (b) H-GTAGA**T**CACT-^L^Lys-NH_2_, or (c) H-GTAGA**T**CACT-NH_2_. The backbone at the **T** position was constructed with the monomer derived from the indicated amino acid.

### 3.2. Duplex Invasion of γ-PNAs

**Figure 8:**
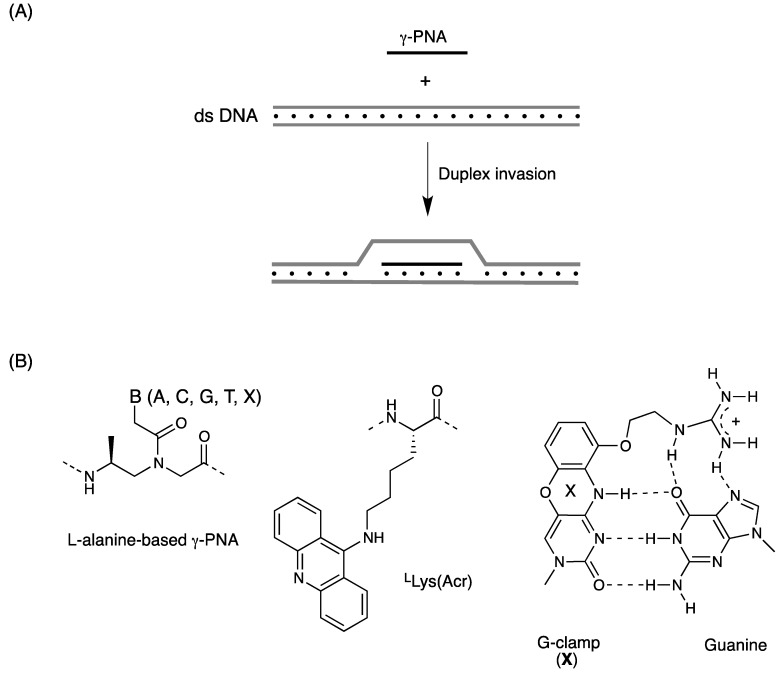
(**A**) Illustration of duplex invasion process of γ-PNA; (**B**) Structures of L-alanine-based γ-PNA, acridine-linked L-lysine residue, and G-clamp-G base pair [75,76,77,78].

**Figure 9:**
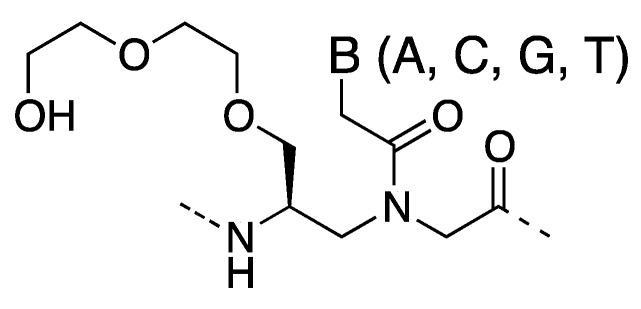
Structure of γ-MiniPEG PNA [60,79].

One of the most remarkable properties of PNA is their ability to recognize some sequences within duplex DNA by strand invasion. Strand invasion proceeds by three distinct mechanisms depending on the DNA target and the PNA sequence: triplex invasion [1], double-duplex invasion [36], and duplex invasion [71,72,73,74]. DNA targets for triplex invasion are basically limited to homopurine sequences. Double-duplex invasion mode can target a wider range of sequences than triplex invasion, still limited to AT-rich sequences. In principle, simple duplex invasion mode does not have sequence limitations; however, duplex invasion complexes are much less stable than the others. As a result, duplex invasion proceeds only when targeting topologically constrained supercoiled plasmid DNA [72] or transcription start sites that offer a single-stranded region [11]. To target any site within the human genome, new mixed-sequence PNAs that can invade canonical B-form duplex DNA need to be developed. γ-PNA appears to fulfill this purpose. Ly *et al*. have reported that mixed-sequence L-alanine-based γ-PNA decamers can invade linear duplex B-form DNA with the assistance of terminal acridine or modified nucleobase (G-clamp) (Figure 8, Table 4) [75,76]. Although strand invasion is usually performed under low salt conditions, incorporation of G-clamp nucleobases enabled invasion even at physiological ionic strength [77]. Moreover, γ-PNAs with a length of 15–20 nucleotides could invade duplex DNA without the need to attach any ancillary agents to PNAs [78]. Recently, MiniPEG-containing γ-PNA was reported that possessed further improved DNA binding properties by eliminating nonspecific binding (Figure 9) [60,79].

**Table 4:** γ-PNA oligomers used for duplex invasion [75,76,77,78] ^a^.

PNA sequences	*T*_m_ (°C)	Duplex invasion	Reference
PNA1: H-^L^Lys-GACCACAGAT-^L^Lys-NH_2_	59	-	[75]
PNA2: H-^L^Lys-**GACCACAGAT**-^L^Lys-NH_2_	~90	-	
PNA3: H-^L^Lys-**GACCACAGAT**-^L^Lys(Acr)-^L^Lys-NH_2_	~90	+	
PNA4: H-^L^Lys-**GAXCACAGAT**-^L^Lys-NH_2_	n.d.	+	[76,77]
PNA5: H-^L^Lys-**GAXCAXAGAT**-^L^Lys-NH_2_	n.d.	+	
PNA6: H-^L^Lys-**GAXXAXAGAT**-^L^Lys-NH_2_	n.d.	+	
PNA7: H-^L^Lys-**GACCACAGATCTAAG**-^L^Lys-NH_2_	>95	+	[78]
PNA8: H-^L^Lys-**TATGAGACCACAGATCTAAG**-^L^Lys-NH_2_	>95	+	

^a^ Bold letters indicate γ-modified backbone units. **X** is G-clamp. n.d.: not determined.

### 3.3. Cell Internalization of γ-PNAs and Related Modified PNAs

**Figure 10:**
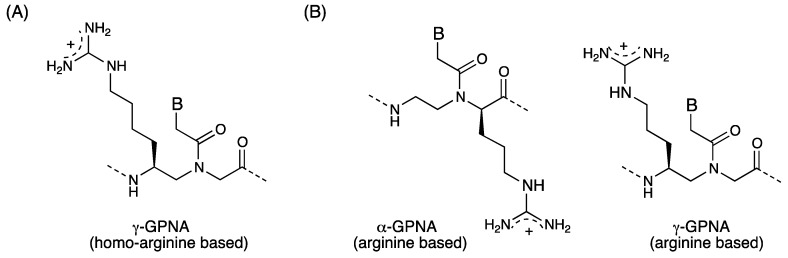
Structures of γ-GPNAs [59,80].

As described in Section 2.2, the ability of α-GPNAs to traverse the cell membrane, and bind to target RNAs in living cells without significant toxicity made them promising agents for antisense applications; however, the high cost associated with monomer synthesis hampered large-scale preparation for clinical testing. Ly *et al*. have reported the synthesis of γ-GPNA, the second-generation GPNA, which was prepared from inexpensive Boc-L-Lysine (selected based purely on cost) and had a homo-arginine side chain at the γ-position (Figure 10A) [59]. Stabilization of PNA-DNA duplexes by incorporation of γ-GPNA units was significant compared to α-GPNAs. Alternate backbone spacing was more effective to enhance thermal stability than the consecutive arrangement. The authors attributed the enhancement of thermal stability to conformational preorganization, not to electrostatic interactions between guanidinium and phosphate groups because of the lack of salt dependence of Δ*T*_m_. A fully alternate γ-GPNA decamer was taken up by HeLa cells and the uptake efficiency was comparable to that of the TAT transduction domain. As in the case of α-GPNAs, γ-GPNAs localized in the ER. The authors attributed the improved cellular uptake of γ-GPNA to their helical conformation.

Recently, inhibition of micro-RNA by GPNAs has been reported by Manicardi *et al*. [80]. Anti-miR-210 activity of PNAs in leukemic K562 cells was examined using a series of 18-mer PNAs: unmodified PNAs, PNAs conjugated with arginine octamer and modified PNAs containing eight units of α- or γ-GPNA monomers. γ-GPNA used by this group carried an arginine side chain (Figure 10B). Two types of placement of GPNA monomers in the sequence were used: alternate spacing and consecutive placement of GPNA units at the *N*-terminus. All modified PNAs were efficiently internalized and the fluorescence of the labeled PNAs was mainly cytoplasmatic. It is noteworthy that uptake of α-GPNAs was resistant to serum. The best anti-miR-210 activity was exhibited by γ-GPNA with consecutive placement. The authors pointed out that overall anti-miR activity of GPNAs is a combination of cellular uptake and RNA binding. The substituents in the PNA backbone play a role not only in cellular uptake, but also in the mechanism of miR recognition and inactivation.

They also compared the DNA binding affinity and sequence selectivity of the PNA containing three adjacent α-GPNA monomers with those of the corresponding γ-GPNA [81]. The γ-GPNA exhibited higher *T*_m_, whereas α-GPNA exhibited higher sequence selectivity. The difference in sequence selectivity between α-GPNA and γ-GPNA was ascribed to the position of the side chains. The side chain in the α-GPNA monomer was attached to the more rigid glycine moiety of the PNA backbone, whereas that of the γ-GPNA monomer was placed in the more flexible aminoethyl moiety. This rigidity may enhance steric repulsion between the side chains when hybridized with mismatched DNA.

**Figure 11:**
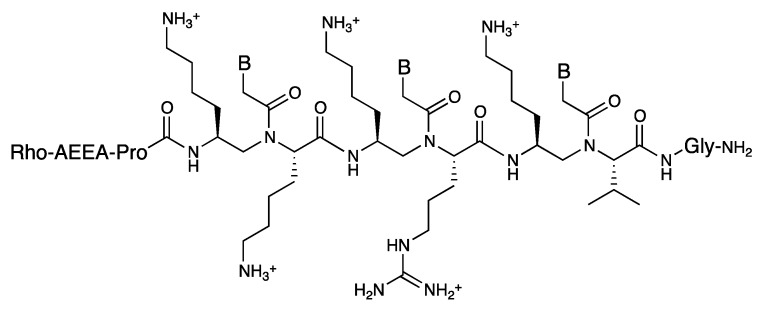
Structure of the modified PNA containing the embedded NLS sequence [82].

The same group reported modified PNA with a nuclear localization signal (NLS) sequence embedded in the PNA backbone (Figure 11) [82]. The PNA was synthesized by a submonomeric strategy and its cellular uptake by rhabdomyosarcoma cells (RH30 cells) was investigated. Both the modified PNA and the NLS peptide were internalized into RH30 cells and localized to the nuclei, whereas unmodified PNA was not detected in the nuclei. It is known that the NLS peptide is transported to the nuclei by an importin-mediated mechanism. Based on the similarity of the localization patterns, it was assumed that the modified PNA could interact with importin. Although PNA was originally developed as a nucleic acid mimic, the authors intend to explore its potential as a peptide mimic.

**Figure 12:**
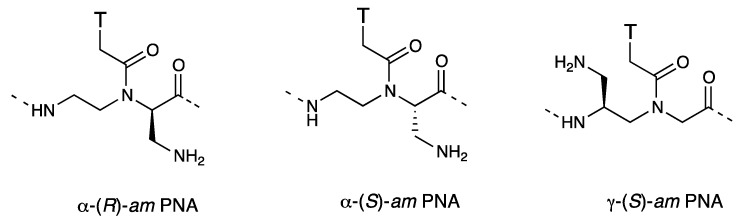
Structures of aminomethyl-PNAs [83,84].

Ganesh *et al*. reported the synthesis of chiral PNAs (*am*-PNAs) with cationic aminomethyl groups at the α- or γ-position of the PNA backbone (Figure 12) [83,84]. The *am*-PNAs formed more stable PNA-DNA duplexes than the unmodified PNA and the order of stabilization was γ-(*S*)-*am* PNA > α-(*R*)-*am* PNA > α-(*S*)-*am* PNA. The *am*-PNAs could traverse the cell membrane of HeLa cells and localized into the nucleus. The order of cellular uptake efficiency of *am*-PNAs was again γ-(*S*) > α-(*R*) > α-(*S*).

## 4. β-PNA

Although some cyclic PNA analogues contain a chiral center at the β-position [85,86,87,88,89,90,91,92], PNAs with a single substituent only at the β-position (β-PNAs) have not been synthesized until recently. In 2011, Sugiyama *et al*. reported the first β-PNA bearing a methyl group at the β-position [93]. Since the β-position of the PNA backbone corresponds to the C4' of the deoxyribose moiety of DNA and the C4' is a chiral carbon atom, the incorporation of a substituent at the β-position was expected to significantly affect the conformation and the DNA binding properties of PNA oligomers.

**Scheme 4:**
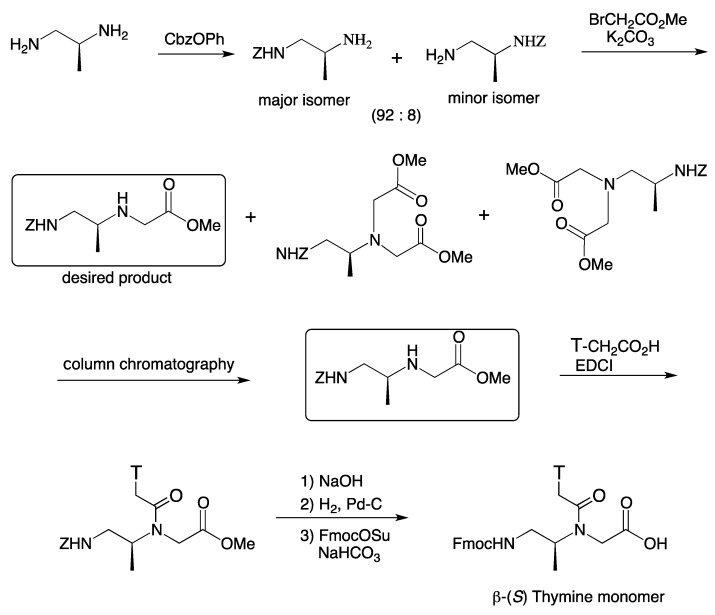
Synthesis of β-(*S*)-Methyl PNA thymine monomer [93].

A PNA monomer possessing a methyl group at the β-position was designed and both enantiomers, β-(*S*)- and β-(*R*)-configurations, were prepared. Scheme 4 illustrates the synthesis of β-(*S*) thymine monomer. Using CbzOPh, (*S*)-1,2-diaminopropane derived from L-alanine was selectively mono-protected on the primary group located on the primary carbon atom in the presence of the primary amino group located on the secondary carbon. Although the requisite compound was obtained as the major isomer (major isomer: minor isomer = 92:8), it could not be separated from the minor isomer by the usual chromatographic methods. Thus, the mixture was directly subjected to alkylation with excess methyl bromoacetate. The minor isomer was completely dialkylated under the reaction condition and became chromatographically separable and thus, the Z-protected β-methyl PNA backbone was obtained. Coupling of the thymin-1-ylacetic acid was accomplished with EDCI. Alkaline hydrolysis, catalytic hydrogenation, and subsequent Fmoc protection with FmocOSu gave the *N*-Fmoc-protected β-PNA monomer. The corresponding enantiomer was also prepared from (*R*)-1,2-diaminopropane.

β-PNA containing three *S*-form chiral units (derived from L-alanine) and unmodified PNA showed similar *T*_m_ values (Table 5). In contrast, the enantiomer β-PNA carrying three *R*-form units (derived from D-alanine) did not bind to DNA. Thus, the stereochemistry of the β-carbon of the PNA backbone was critical to the hybridization ability of PNA and strictly limited to *S*-configuration. This contrasts to α-PNAs in which stereochemistry of the α-carbon arising from a methyl group hardly affected the DNA binding ability.

**Table 5:** Melting temperatures (°C) of β-PNA-DNA duplexes [93] ^a^.

PNA sequence	*T_m_* (°C)
H-GTAGATCACT-^L^Lys-NH_2_	51.4
H-G **T^S^**AGA**T^S^**CAC**T^S^**-^L^Lys-NH_2_	51.0
H-G **T^R^**AGA**T^R^**CAC**T^R^**-^D^Lys-NH_2_	n.d.
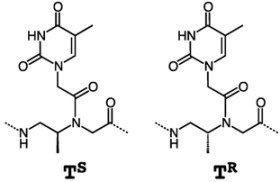	

^a^ Bold letters indicate modified backbone units.

The circular dichroism (CD) spectra of β-PNAs indicated that β-(*S*)-PNA adopted a right-handed helical structure and β-(*R*)-PNA was left-handed; however, the induced right-handed structure of β-(*S*)-PNA did not contribute to the total PNA-DNA duplex stability. The benefit of the induced structure might be abolished by unfavorable steric interactions arising from β-methyl groups in the PNA-DNA hybrid duplex. Since the development of β-PNA is a new, unexplored field, more sophisticated designs may be possible.

## 5. Conclusions

The great potential of PNA has stimulated chemists to develop new PNA analogues with superior properties to unmodified PNA. A large number of modified PNAs have been obtained by modifying the PNA backbone, nucleobases, and the linker connecting a nucleobase to the backbone. As described in this review, introduction of a substituent to the PNA backbone is a simple strategy but has proved fruitful in improving DNA binding properties and the sequence selectivity of PNAs. γ-PNAs seem to be the most potent in terms of DNA/RNA binding affinity. GPNAs provide a solution for the problem of poor cellular uptake of unmodified PNA. The accurate control of intracellular localization of PNAs will be the next challenge.

The synthetic protocol of modified PNAs with a substituent on the backbone is straightforward and therefore the cost of large-scale synthesis is within an acceptable range. Sufficient quantities of various modified PNAs will be available for biological investigations and animal experiments in the near future. Considering the application of PNAs for therapeutic purposes, issues such as metabolic pathways, pharmacokinetics and pharmacodynamics need to be taken into account.
